# Understanding the transmission of *Mycobacterium ulcerans*: A step towards controlling Buruli ulcer

**DOI:** 10.1371/journal.pntd.0009678

**Published:** 2021-08-26

**Authors:** Anthony J. Muleta, Rachael Lappan, Timothy P. Stinear, Chris Greening

**Affiliations:** 1 Department of Microbiology, Monash Biomedicine Discovery Institute, Monash University, Clayton, Australia; 2 Department of Microbiology and Immunology, Doherty Institute for Infection and Immunity, University of Melbourne, Melbourne, Australia; 3 Centre to Impact AMR, Monash University, Melbourne, Australia; University of Surrey Faculty of Health and Medical Sciences, UNITED KINGDOM

## Abstract

*Mycobacterium ulcerans* is the causative agent of Buruli ulcer, a rare but chronic debilitating skin and soft tissue disease found predominantly in West Africa and Southeast Australia. While a moderate body of research has examined the distribution of *M*. *ulcerans*, the specific route(s) of transmission of this bacterium remain unknown, hindering control efforts. *M*. *ulcerans* is considered an environmental pathogen given it is associated with lentic ecosystems and human-to-human spread is negligible. However, the pathogen is also carried by various mammals and invertebrates, which may serve as key reservoirs and mechanical vectors, respectively. Here, we examine and review recent evidence from these endemic regions on potential transmission pathways, noting differences in findings between Africa and Australia, and summarising the risk and protective factors associated with Buruli ulcer transmission. We also discuss evidence suggesting that environmental disturbance and human population changes precede outbreaks. We note five key research priorities, including adoption of One Health frameworks, to resolve transmission pathways and inform control strategies to reduce the spread of Buruli ulcer.

## Author summary

Buruli ulcer is a debilitating skin and soft tissue disease characterised by large ulcerative wounds that are treated with antibiotics or with adjunctive surgery for advanced cases. Found predominantly in West Africa and Southeast Australia, the causative agent is the environmental bacterial pathogen *Mycobacterium ulcerans*. Lack of understanding of transmission pathways, combined with the absence of a vaccine, has hindered efforts to control the spread of *M*. *ulcerans*. Here, in order to identify probable transmission pathways and inform future studies, we review literature linking *M*. *ulcerans* to environmental reservoirs, mammalian hosts, and potential invertebrate vectors. We also summarise factors and behaviours that reduce the risk of developing Buruli ulcer, to inform effective prevention strategies and further shed light on transmission pathways.

## Introduction

Buruli ulcer is a rare but devastating bacterial skin and soft tissue infection caused by *Mycobacterium ulcerans*. It usually presents as cutaneous lesions that necrotise and progress to painless ulcers. Despite being treatable with antibiotics, 31% of cases exhibit severe lesions that can be disabling and stigmatising [1]. Reported in at least 33 countries, the pathogen has been most reported and intensively studied in West Africa and Southeast Australia [2,3]. Strategies for effective control of the disease depend on a clear understanding of the transmission pathways of *M*. *ulcerans*. Yet, despite a body of research spanning more than 80 years examining Buruli ulcer risk factors and prevention methods, the mode(s) of transmission remain unresolved [4,5]. Investigation into *M*. *ulcerans* transmission by tracing clusters and cases back to the source of infection is further complicated by the relative rarity of Buruli ulcer (approximately 3,000 to 4,000 reported global cases annually [1]; less than 2% of annual leprosy case numbers [6]) and a 5-month incubation period [7]. The absence of an effective vaccine against *M*. *ulcerans* further heightens the importance of disease control and prevention in the management of Buruli ulcer worldwide.

**Fig 1:**
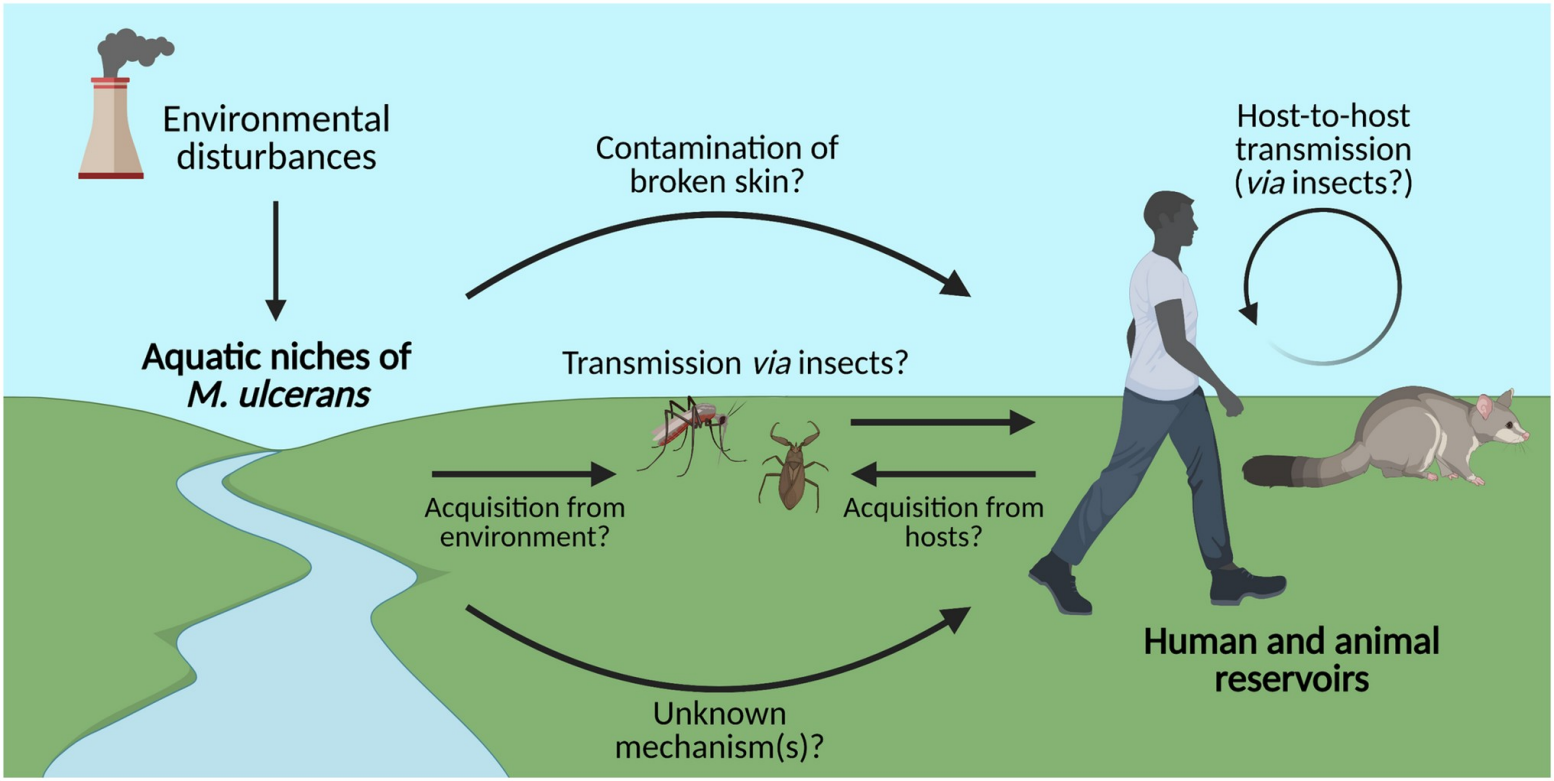
Proposed mechanisms of transmission of *Mycobacterium ulcerans*, the causative agent of Buruli ulcer. The mode(s) of transmission of Buruli ulcer are still uncertain and are likely to differ between West Africa and Southeast Australia. However, hypotheses such as *M*. *ulcerans* contamination via aquatic niches [10], host-to-host transmission between humans and animal reservoirs (including possums) [8], and transmission via insect mechanical vectors (including mosquitoes) [9] have been proposed. These hypotheses are explored in this review. Created with BioRender.com.

Several possible modes of transmission have been proposed (Fig 1), with evidence for these scenarios varying by region, as summarised in Table 1. Generally, *M*. *ulcerans* is considered an environmental pathogen associated with freshwater ecosystems. However, it remains unclear whether the pathogen can be directly transmitted from the environment to humans, for example, from contaminated water, sediments, vegetation, or aerosols, to broken skin [4,5]. Increasing evidence suggests that certain mammals can serve as reservoirs for the pathogen (which we define as hosts or environments in which *M*. *ulcerans* lives and reproduces), while aquatic insects and mosquitoes may serve as carriers and potentially mechanical vectors [8,9]. In this review, we summarise the current evidence surrounding the involvement of known reservoirs, the likelihood of different transmission pathways based on experimental studies and observed risk factors, and how environmental disturbance may be connected to Buruli ulcer outbreaks. Furthermore, we propose directions for future research targeting the resolution of *M*. *ulcerans* transmission to enable clear recommendations for efficient disease control in vulnerable areas.

**Table 1:** Summary of current evidence regarding proposed routes of transmission of *Mycobacterium ulcerans* in West Africa and Southeast Australia.

Route of transmission	West Africa	Southeast Australia
**Contamination of broken skin**	Unknown. Inoculation of *M*. *ulcerans* on to abraded skin does not cause infection in guinea pigs [11]. However, proper hygiene and wound care are associated with reduced odds of developing infection in Africa [12].	
**Breaking of contaminated skin**	Possible. Needle puncture was sufficient to allow *M*. *ulcerans* to enter skin and cause infection in mice [9]. Infection has been reported following breaking of skin via human bite [13,14].	
**Via aquatic environments**	Likely. Environmental *M*. *ulcerans* DNA identical to genetic profiles of local human Buruli ulcer cases [15]. Transmission mechanism unknown.	Possible. Buruli ulcer case numbers increase in rainy seasons [16]. Transmission mechanism unknown.
**Human to human**	Unlikely. Analysis of familial clusters suggests that infections were due to genetic predispositions, rather than human-to-human transmission [17].	Unlikely. Analysis of familial clusters found that family members were infected with different strains of *M*. *ulcerans*, suggesting that human-to-human transmission had not occurred [18].
**Vertebrate to human**	Unlikely. *M*. *ulcerans* has not been recovered from domestic animal samples [19]. *M*. *ulcerans* reported in wild grasscutters [20,21].	Unknown. Possums are hosts of *M*. *ulcerans* strains also isolated from human patients [8], part of same transmission chain.
**Aquatic insect vectors**	Possible. *M*. *ulcerans* DNA has been recovered from African insects in endemic areas [22]. Insects have been shown to carry *M*. *ulcerans* and transmit to mice [23].	Unknown. Studies have not been conducted.
**Mosquito vectors**	Unlikely. *M*. *ulcerans* has not been recovered from mosquito populations [24], and bacilli are not maintained through mosquito life cycle [25].	Likely. Very strong geographical correlation between *M*. *ulcerans* detection in mosquito populations and human Buruli ulcer cases [26]. Mosquito bites can facilitate *M*. *ulcerans* infection in mice [9].

## Inferences from phylogenomic studies

Examining the evolution and spread of *M*. *ulcerans* across different regions can help to elucidate the transmission and movement of this pathogen between environments and reservoirs. Comparative genomic studies indicate that *M*. *ulcerans* likely evolved from an ancestor of *Mycobacterium marinum* [27,28]. *M*. *ulcerans* and *M*. *marinum* are both bacteria associated with aquatic environments that cause opportunistic animal and human infections [10,29]. Yet, while their core genomes share 97% nucleotide identity [28], the *M*. *ulcerans* genome contains various signatures of a recent evolutionary bottleneck, characterised by an increased proportion of pseudogenes and substantial chromosomal deletions [30]. Through deletions and mutations, *M*. *ulcerans* has lost various genes required for survival in diverse aquatic habitats, for example, those associated with anaerobic energy conservation and light-dependent pigment biosynthesis [31]. This suggests that *M*. *ulcerans* has become a more specialist bacterium that primarily or solely grows in host-associated niches [30,32]. Importantly, *M*. *ulcerans* has also acquired the virulence plasmid pMUM001 that confers the ability to synthesise the cytotoxic and immunosuppressive toxin mycolactone and thus cause more serious ulcerative disease than *M*. *marinum* [28].

Further bacterial evolution and dissemination events gave rise to the *M*. *ulcerans* lineage primarily responsible for causing Buruli ulcer today [28,33]. Genomic analysis of West African strains of *M*. *ulcerans* suggests that the bacterium was introduced into Africa at least twice, first circa 68 BC and second during the 1800s [34]. Both introductions were highly geographically localised, but there was significant proliferation and expansion concurrent with the human colonisation of these regions [34]. Through phylogenetic, spatial, and temporal analyses, it has been shown that *M*. *ulcerans* migrated through Victoria, Australia, following an increase in human population size [35]. *M*. *ulcerans* and Buruli ulcer disease appear to have been introduced into east Australia in the early 1800s, before spreading westwards in the 1980s towards more populous cities, such as Melbourne, Victoria [35]. Similar analyses have shown that cases of Buruli ulcer rose in regions of central Africa following European colonisation [36]. In West Africa, the different genetic lineages of *M*. *ulcerans* are generally localised to specific regions and areas, which supports the possibility of an aquatic reservoir restricting bacterial movement between these regions [34,37–39]. Yet, at smaller scales, different genotypes of *M*. *ulcerans* in Africa have been found in areas dominated by another genotype [40], demonstrating the ability of the pathogen to mobilise more locally, potentially via a vector. The emergence patterns in both continents suggests that *M*. *ulcerans* may be able to mobilise and spread along gradients of human population and activity, though increased detection may also be a consequence of increased disease presentation in people.

## Methods for *Mycobacterium ulcerans* surveillance

Resolving the transmission pathways of *M*. *ulcerans* depends on being able to detect and isolate the pathogen from diverse sample types. Most surveillance studies of *M*. *ulcerans* rely on quantitative PCR (qPCR)-based detection of specific markers. For clinical diagnosis, TaqMan qPCR assays targeting the multicopy IS*2404* insertion sequence (with 213 copies in the *M*. *ulcerans* genome [30,41]) remain the gold standard for detecting Buruli ulcer due to their high specificity, high sensitivity, and rapid turnaround time [42,43]. For environmental surveys, a multiplex qPCR assay is typically used to simultaneously detect IS*2404*, IS*2606* (another multicopy insertion sequence), and ketoreductase-B (KR-B) DNA (required for mycolactone biosynthesis). Using these markers, *M*. *ulcerans* DNA has been reliably detected by PCR or qPCR across diverse waters, soils, invertebrates, vertebrates, and plant-associated samples [8,42,44–47]. However, as previously noted [5], the information gained from such detection is limited. Most importantly, it is difficult to discern whether the small quantities of DNA often detected are relevant for resolving transmission cycles. Through such methods alone, it is also impossible to determine whether the pathogen is viable in different samples (in contrast to cultivation) or comes from the same lineage as infected patients (in contrast to genomics and amplicon sequencing).

At present, the challenges associated with cultivating *M*. *ulcerans* mean that we lack a strong understanding of its viability and population structure in environmental and animal samples. The pathogen typically takes 2 to 3 months to grow on an agar plate and will be rapidly outgrown by bacterial and fungal contaminants if decontamination steps aren’t taken [48]. Moreover, its abundance is often too low in environmental or animal samples to be reliably cultivated [49]. As a result, there are few examples of *M*. *ulcerans* being isolated in axenic cultures from aquatic sources [49,50]. However, there may be potential to improve cultivation methods to better select and differentiate *M*. *ulcerans*. For example, a recent study combined a selective medium (Middlebrook 7H10 agar with chlorhexidine decontamination) and a differential readout (F_420_-based autofluorescence) to cultivate *M*. *ulcerans* from the faeces of wild grasscutters (greater cane rats; *Thryonomys swinderianus*) [20]. There is also the potential to leverage recent advances in culture-independent sequencing, including metagenomic mapping and assembly or tiled amplicon sequencing, to better resolve population structure and potential transmission dynamics of *M*. *ulcerans* between human, animal, and environmental sources.

## Humans as reservoirs of *Mycobacterium ulcerans*

The most frequently reported host of *M*. *ulcerans* is humans, although direct human-to-human transmission has not been demonstrated or documented and is considered an unlikely route in endemic regions of both Africa and Australia. Although one case of Buruli ulcer was reported following a human bite in Benin, it is thought that the pathogen entered the wound from an environmental rather than oral source [13,14]. In the absence of confirmed transmission and experimental studies, familial and genetic analyses of infected individuals and causative strains of *M*. *ulcerans* can provide insight into the possibility of transmission between humans. A case–control study conducted in Benin found that individuals were five times more likely to develop Buruli ulcer if a family member had previously contracted the disease; however, this was attributed to genetic predispositions to infection rather than familial transmission, though cases were not genotypically matched to controls [17]. Several alleles and genetic polymorphisms have been predicted to confer an increased susceptibility to developing symptomatic Buruli ulcer, which may contribute to the emergence of familial clusters of this disease [51,52]. A more recent study conducted in Victoria, Australia examined familial clusters of Buruli ulcer infections and found that *M*. *ulcerans* isolates differed in genotype between family members, resolving that transmission between family members had not occurred [53]. This study also found that family members were diagnosed at similar times (shorter than the median incubation time of approximately 4.5 months), suggesting they acquired infection from a common environmental source within weeks of each other [53]. The absence of evidence for human-to-human transmission suggests that *M*. *ulcerans* is not transmitted between humans, implicating environmental and other potential routes of transmission.

## Environmental reservoirs of *Mycobacterium ulcerans*

To understand the enigma of Buruli ulcer transmission, consideration of the environmental reservoirs in which *M*. *ulcerans* is found is critical, as these may overlap with human activity and present opportunities for infection. *M*. *ulcerans* is considered an environmental pathogen, given evidence from epidemiological studies from both Southeast Australia and West Africa that Buruli ulcer primarily occurs in areas with swamps and slow-flowing water, and the lack of evidence of human-to-human spread [10,15,17,53–55]. Indeed, fine spatial modelling suggests that proximity to waterbodies and related factors were the strongest predictors of Buruli ulcer occurrence and *M*. *ulcerans* suitability [56,57]. Likewise, from a social perspective, residents in the Ivory Coast (a Buruli ulcer–endemic region of Africa) also associate unclean water with acquiring Buruli ulcer [58].

Such inferences are supported to some extent by molecular evidence on the distribution of *M*. *ulcerans* in aquatic habitats. Multiple studies have detected *M*. *ulcerans* DNA in aquatic ecosystems, such as stagnant water sources, in West Africa and French Guiana [59–61]. For example, Bratschi and colleagues tested samples from shallow water holes in Cameroon over a period of more than 2 years and found that *M*. *ulcerans* DNA was consistently present over the entire duration of the study [10]. An important feature of this study was that the sample collection sites were water sources used by villagers for bathing and washing, supporting the hypothesis of environmental transmission through aquatic reservoirs. Environmental detection of *M*. *ulcerans* DNA in West Africa is positively correlated with the incidence of Buruli ulcer in an area, supporting the possibility of an environmental transmission route [62]. *M*. *ulcerans* is also detected more frequently during wet (rainy) seasons, in endemic regions of both Africa and Australia, with Buruli ulcer case numbers increasing during these periods [16,54,63,64]. The increased environmental load of *M*. *ulcerans* reported during these rainy seasons likely contributes to the observed increase in case numbers [63]. This is reminiscent of the transmission of leptospirosis, which increases after flooding or heavy rain [65]. Other than water bodies, *M*. *ulcerans* is known to persist for extended periods in various other environments [66,67]. *M*. *ulcerans* DNA has additionally been detected in West Africa in diverse aquatic plant species [15,46,68,69] and animals, including snails, fish, and amphibians [47,70,71], which may contribute to hosting and maintaining *M*. *ulcerans* in an aquatic environment. *M*. *ulcerans* DNA has also been detected in amoebae, though at a low prevalence based on PCR screening only [72,73].

An important caveat is that most environmental surveys solely rely on qPCR-based detection of *M*. *ulcerans* DNA. As noted above, such approaches do not provide information on whether the pathogen is viable, transmissible, and shares genetic history with human isolates in samples it is detected from. Some studies have partially genotyped samples (e.g., through variable number of tandem repeats (VNTR) profiling) but have not precisely resolved phylogenetic relationships between *M*. *ulcerans* in human and environmental sources [55,74,75]. Also in counterpoint, a study in Ghana by Pileggi and colleagues found a negative association between the wetness index of an area and the odds of *M*. *ulcerans* DNA presence, seemingly contradicting the accepted paradigm of *M*. *ulcerans* as an aquatic pathogen [76]. However, aquatic sites were only sampled over a single day and selected sites were restricted to large bodies of water, excluding many smaller niches. It also remains unclear whether *M*. *ulcerans* can be directly transmitted from water sources independently of a vector. Studies in guinea pigs found that bacteria inoculated onto abraded skin did not lead to infection [11]. Rather, disease in guinea pigs was only observed after subcutaneous bacterial inoculation, a finding reinforced from infection studies in mice that showed infection was only established after needle puncture (or mosquito blood feeding) [9]. It is possible that infections may be acquired directly from the environment via deeper cuts and more exposed wounds, similar to leptospirosis where existing lacerations increase the risk of infection from a contaminated environment [65], though experimental studies demonstrating this in Buruli ulcer are lacking.

## Aquatic insects in *Mycobacterium ulcerans* transmission

Given *M*. *ulcerans* is linked with lentic systems and has signatures of a host-adapted lifestyle, it has been proposed that water bugs and other aquatic insects serve as vectors. Several studies have focused on elucidating the role of aquatic insects in the transmission of *M*. *ulcerans* in West Africa, though corresponding research efforts have not been conducted in Australia. The association of *M*. *ulcerans* DNA with aquatic insects has been confirmed in endemic regions of Africa, particularly Benin [22]. In addition, a recent model of aquatic insect distribution in West Africa predicted that insects likely to be involved in the transmission of Buruli ulcer were those which had adapted to environments of two highly affected regions, Ghana and Cameroon [77]. Also consistently, aquatic insects are one of the main perceived causes of Buruli ulcer by residents in the Ivory Coast [58], and receiving insect bites near a river has recently been identified as a risk factor for developing Buruli ulcer in Togo [78].

A 2014 case report describes a 6-year-old girl in Benin who presented with ulcers and recalled being bitten by an aquatic insect of the Belostomatidae (giant water bug) family at the site of ulceration [79]. This occurred near an aquatic environment, so it is not clear whether the source of infection was the insect vector or if the bite simply facilitated entry from the environment. No other direct evidence of insect transmission to a human has been reported to date. Experimentally, African aquatic insects of the Belostomatidae family (*Appasus* spp., *Diplonychus* spp.) have been shown to carry *M*. *ulcerans* and transmit bacteria to their larvae [80]. A major study isolated an *M*. *ulcerans* strain from water striders (Gerridae family, *Gerris* spp.); this strain shared genotypic and phenotypic features to human isolates and caused severe infection in a mouse footpad model [49]. Notably, this is the only report of successful axenic cultivation of *M*. *ulcerans* from an environmental sample worldwide. Naucoridae (creeping water bugs) can also carry *M*. *ulcerans* and, in an experimental model, transmit the bacteria to mice by biting, further supporting the possibility of environmental transmission via insects [23,81].

Despite the above evidence, aquatic insects have been described as “unlikely vectors” for *M*. *ulcerans* on several grounds [82]. A field study in Ghana collected over 22,000 aquatic insects and found no difference in numbers of insect communities positive for *M*. *ulcerans* between endemic and nonendemic sites (less than 2% positive across the study), which questions the relationship of these insects with carriage and transmission of Buruli ulcer in West Africa [82]. There is also conflicting evidence whether the abundance of these insects correlates with Buruli ulcer incidence [82–84]. Moreover, Belostomatidae and Naucoridae rarely bite people and their trophic ecology is atypical of disease vectors [5,82]. Mathematical modelling has also shown that spatial and temporal variations of Buruli ulcer incidence are better explained by environmental rather than water bug transmission [85]. A systematic review further critiques the evidence for the role of aquatic insects and proposes establishing more rigorous criteria for vector incrimination [5].

## Mosquitoes in *Mycobacterium ulcerans* transmission

Molecular studies have implicated mosquitoes as potential mechanical vectors for *M*. *ulcerans* in Australia, where *M*. *ulcerans* DNA has been recovered from mosquito populations in endemic areas [26,86]. In particular, Lavender and colleagues collected data simultaneously with human Buruli ulcer case data in Victoria and found a very strong positive dose–response relationship between *M*. *ulcerans* DNA detection in mosquitoes and Buruli ulcer cases in each town (r = 0.99, *p* < 0.001) [26]. Specifically, mosquitoes were more likely positive for *M*. *ulcerans* DNA in Point Lonsdale, the town with the greatest number of cases in the study. A case–control study in Southeast Australia found that a history of mosquito bites was associated with an increased risk of Buruli ulcer, while use of insect repellents and long-sleeved clothing was associated with a reduced risk of infection [87]. A role for mosquitoes in the spread of Buruli ulcer is supported further by the fact that Buruli ulcer lesions develop preferentially on extremities of limbs, which are often exposed and susceptible to mosquito bites [88]. It has also been found in Victoria, Australia that the majority of Buruli ulcer infections occur in the warmer months, when the abundance of mosquitoes is highest and individuals are more likely to wear clothing that leaves skin and limbs exposed to mosquitoes and other biting insects [88]. Contrastingly, an environmental survey of mosquitoes in Queensland, Australia found that while mosquitoes were positive for genetic markers of the pathogen, genetic analysis revealed divergence between human pathogenic strains and those isolated from mosquitoes [89]. Further genetic comparisons of clinical and mosquito-associated strains of *M*. *ulcerans* may clarify the role mosquitoes play in transmission in various endemic regions of Australia.

A recent study by Wallace and colleagues demonstrated that Australian mosquito bites (*Aedes notoscriptus*) facilitate *M*. *ulcerans* infection in a mouse tail model [9]. Mouse tails were coated with *M*. *ulcerans* and subjected to mosquito biting for 20 minutes. This was sufficient to cause infection, demonstrating that mosquitoes can facilitate the entry of *M*. *ulcerans* through the skin. However, it remains unclear whether mosquitoes can cause infection in natural settings or in humans, whether by providing an entry route for the bacterium when it is already present on the skin, or by acting as a mechanical vector by providing both the entry route and the bacterium. This study also found that *M*. *ulcerans* has a low infectious dose, which is a commonly observed feature of vector-borne diseases [9]. Other experimental studies suggest that mycolactone may serve as a potential attractant for mosquitoes, thereby facilitating *M*. *ulcerans* transmission [90,91]. Ultimately, further experimental and observational studies are needed to establish vector competence.

In contrast, the evidence for mosquito transmission is lacking in West Africa. Environmental surveillance studies have failed to detect both *M*. *ulcerans* bacteria and DNA in mosquito populations of endemic areas of Africa, suggesting that mosquitoes do not play a major role in the carriage or transmission of *M*. *ulcerans* as they appear to in some areas of Australia [22,24]. Additionally, experimental studies have shown that while African mosquito larvae (*Anopheles gambiae*) can ingest and host *M*. *ulcerans* during larval developmental stages, bacilli are absent by the adult stage where they would be transmitted to offspring, so biological transmission via mosquitoes appears to be unlikely in West Africa [24,25]. Due to the long incubation time of Buruli ulcer, it is difficult to trace an infection back to a brief event such as a mosquito bite. More rigorous surveillance of endemic regions and mosquito activity in West Africa is needed before any conclusions can be made. Currently, the contrasting evidence between Southeast Australian and West African studies suggests that the mode(s) of transmission of Buruli ulcer may be different, with mosquitoes playing a more significant role in Australia. This could be a consequence of different bacterial strains, mosquito species/populations, environmental factors, and/or social behaviours between the two continents. Direct comparisons of Australian and African mosquito species or *M*. *ulcerans* strains may further resolve this difference.

## Mammalian reservoirs of *Mycobacterium ulcerans*

In addition to humans, several other mammals have also been shown to become infected with *M*. *ulcerans* [92]. Overall, evidence for animals as hosts of *M*. *ulcerans* is stronger for Australian animals than for African animals, which may reflect geographical differences in *M*. *ulcerans* lineages and/or routes of transmission. In Point Lonsdale, a Buruli ulcer–endemic area of Southeast Australia, Fyfe and colleagues found that over one-fifth of Australian native possums (*Pseudocheirus peregrinus* and *Trichosurus vulpecula*) were positive for *M*. *ulcerans* DNA and shed bacterial DNA in their faecal pellets, and several showed lesions characteristic of symptomatic Buruli ulcer [8]. This high prevalence, especially in comparison to observations in other wildlife, points to possums as important reservoirs of *M*. *ulcerans* [92,93]. An isolate was also obtained from a ringtail possum, and subsequent whole genome sequencing revealed that the isolate was virtually identical to human clinical isolates of the same endemic region (differing by only two SNPs), indicating that possums and humans were part of the same transmission chain [8].

Case reports of Buruli ulcer have also been described in a number of other animals in endemic regions of Australia, including koalas [94,95], horses [96], dogs [97], a cat [98], and alpacas [99], where swabs from lesions or ulcers were positive for *M*. *ulcerans* by PCR. These findings highlight the importance of *M*. *ulcerans* as both a human and an animal pathogen, though provide only incomplete evidence for infection in each of these species. Recently in Australia, bandicoots have been investigated as hosts of *M*. *ulcerans*, though studies conducted to date have only identified a total of four faecal samples containing *M*. *ulcerans* DNA [89,100]. One bandicoot has been reported presenting with lesions characteristic of Buruli ulcer, though swabs of these lesions were all negative for *M*. *ulcerans* [100], and local veterinarians did not report any Buruli ulcer cases in bandicoots. Furthermore, molecular detection in faecal samples may be indicative of exposure to *M*. *ulcerans* rather than infection.

In contrast, there does not appear to be an equivalent vertebrate host in endemic regions of Africa. Grasscutters have recently been proposed as potential hosts of *M*. *ulcerans* in endemic regions of Africa. In the Ivory Coast, almost 20% faecal samples and 80% spleens surveyed were positive for *M*. *ulcerans* DNA, though it is likely that prevalence was overestimated as the criteria for a positive sample in this study were less rigorous than many other studies (one of the IS*2404*, IS*2606*, and KR-B *M*. *ulcerans* genetic markers positive by PCR analysis, rather than all three simultaneously) [21]. An *M*. *ulcerans* isolate has also been cultivated from grasscutter stool, though was not genotypically or phenotypically characterised [20]. Thus, it remains uncertain what role these animals play in pathogen ecology. In contrast to possums, Buruli lesions have not been observed in grasscutters. In most cases, rodents likely ingest *M*. *ulcerans* while consuming aquatic plants without developing systemic infections [101,102]. There is minimal evidence that other animals in West Africa may be hosts of *M*. *ulcerans*. One case study in Benin reported a goat and a dog each presenting with lesions positive for *M*. *ulcerans* DNA, though this is the only report of infection in such animals in Africa [103]. Tobias and colleagues conducted a large-scale study of domestic animal faecal samples in endemic villages in Ghana and did not recover a single positive sample, indicating that it is unlikely that domestic animals play a role in harbouring *M*. *ulcerans* in West Africa [19]. Likewise, a survey of 565 small mammals trapped in Benin did not identify *M*. *ulcerans* [104].

## Environmental disturbance precedes Buruli ulcer outbreaks

Several studies have reported that Buruli ulcer often follows environmental disturbance. Deforestation and a lack of forested land cover have been associated with *M*. *ulcerans* emergence in several regions of West Africa [76,105–107]. Specifically in Ghana, Buruli ulcer incidence is positively associated with urbanisation and mining [105,106]. Similar trends have been observed in the Ivory Coast, where areas in the vicinity of man-made dams and cultivated crop fields were found to be high-risk areas for contracting Buruli ulcer [108,109]. In addition, the emergence of *M*. *ulcerans* has recently been reported in Cameroon as a result of the damming of the Mapé river [110]. Fewer studies on environmental factors facilitating *M*. *ulcerans* emergence have been conducted in Australia. However, a large, localised outbreak in Phillip Island was strongly linked to the construction of a golf course and wetland [111,112]. Based on PCR data, both the golf course irrigation system and wetland were highly contaminated with *M*. *ulcerans* [45,113], with a strong correlation observed between inferred pathogen numbers and reported Buruli ulcer cases observed both spatially and temporally. It has been proposed that *M*. *ulcerans* was transmitted to humans from either the irrigation system through aerosolised droplets or from the wetland via an insect vector [5,45].

These various disturbances to the environment may contribute to the emergence of *M*. *ulcerans* in several ways. As discussed earlier, given *M*. *ulcerans* is primarily found in and adapted to aquatic niches, human activities may create appropriate aquatic environments where *M*. *ulcerans* and potentially their vectors can thrive [16]. Additionally, both increased interactions of humans with aquatic environments and increased proximity of settlements to waterways create opportunities for infections to occur. A study by Morris and colleagues found that human-driven land use changes and deforestation had ecologically significant impacts on the abundance of potential animal hosts of *M*. *ulcerans* within aquatic ecosystems in French Guiana, subsequently increasing the bacterial load within an ecosystem and potentially facilitating transmission to humans via these hosts [114]. Specifically, these environmental disturbances impacted freshwater food webs, causing complex downstream effects in trophic ecology that together favour the emergence and transmission of *M*. *ulcerans*. Overall, human interventions generally diminish local biodiversity and often compromise animal health, creating more favourable conditions for *M*. *ulcerans* to cause infection and potentially vectors to thrive [115,116]. Given the rarity and uncertainty of Buruli ulcer data, environmental and landscape factors such as those described above could be used to predict *M*. *ulcerans* presence, emergence, and potential high-risk areas for Buruli ulcer, which may assist in preventing environmental transmission to humans [105]. Previous reviews provide additional perspectives on the effects of environmental disturbance and potentially global change in *M*. *ulcerans* ecology [5,117].

## Strategies for controlling Buruli ulcer

Given our understanding of the mode(s) of transmission of Buruli ulcer remains low, control of this disease is difficult as specific transmission pathways cannot be interrupted. Rather, evidence taken directly from prevention studies can inform effective strategies for disease control and further elucidate transmission pathways. Several prevention case–control studies conducted in Buruli ulcer–endemic regions of Africa have explored factors that reduce the risk of contracting Buruli ulcer (Table 2), providing insight into potential transmission routes. Contact with water sources in various settings is consistently associated with increased odds of infection, while reducing contact with these environments is protective [118–121]. This is especially important in agricultural settings where individuals may be working in these areas for long periods of time. The use of adequate waterproof protective equipment and sleeved clothing reduces these risk factors and, therefore, the likelihood of developing an infection. A similar approach is recommended to reduce the risk of leptospirosis infection, which is also associated with flooding, wet environments, and existing wounds as potential entry points for infection [65,122]. There is also the potential to develop physical interventions to reduce contact with environmental water sources. For example, a recent study showed a strong negative correlation between the introduction of new wells and the incidence of Buruli ulcer in Benin [123]; an associated case–control study showed that regular use of water from the wells (for washing, bathing, drinking, or cooking) was a protective factor against infection [123].

**Table 2:** Summary of activities and behaviours that influence odds of developing Buruli ulcer infection. Data were taken from case–control studies conducted in regions of Africa endemic for Buruli ulcer. OR refers to the odds of developing Buruli ulcer in cases compared to noninfected controls for “risk factors” and the odds of not developing Buruli ulcer in noninfected individuals compared to infected cases for “protective factors”.

Factor	OR	95% Confidence Interval	Reference
***Risk factor***			
Agricultural contact with surface water	6.3	1.8–21.9	N’Krumah et al. [118]
Recreational contact with surface water	5.7	1.6–20	Pouillot et al. [119]
Washing/bathing in surface water	7.5	2.0–27.8	N’Krumah et al. [118]
	6.9	1.4–34.7	Landier et al. [12]
Absence of protective clothing during agricultural activities	18.5	5.2–66.7	N’Krumah et al. [118]
	15	4.2–58	Pouillot et al. [119]
Receiving insect bites near a river	7.8	1.5–41.2	Maman et al. [78]
Report scratching wounds after insect bites	2.7	1.4–5.4	Landier et al. [12]
Treating wounds with adhesive bandages	6.4	2.2–19	Pouillot et al. [119]
***Protective factor***			
Infrequent contact with flowing water	2.1*	1.4–3.3	Nackers et al. [120]
Washing, bathing, drinking, or cooking with well water	10*	2.3–25	Degnonvi et al. [123]
Footwear use	6.7*	3.4–13	Tomczyk et al. [124]
Regular use of bed nets	2.6	1.2–6.0	Pouillot et al. [119]
	2.5*	1.1–5	Landier et al. [12]
Regularly washing clothing	5.1	1.5–17	Pouillot et al. [119]
Using rubbing alcohol on wounds	2.2	1.0–4.6	Pouillot et al. [119]
Use of soap and good general hygiene	10*	3.3–33	Landier et al. [12]
	2.4*	1.5–4.0	Nackers et al. [120]
	4.0*	2.7–5.9	Nackers et al. [120]
Secondary education or above	3.6	1.3–9.8	Pouillot et al. [119]
Good knowledge of risks that may result in Buruli ulcer	3.3*	1.3–10	N’Krumah et al. [118]
Good knowledge of causes of Buruli ulcer	10*	3.3–50	N’Krumah et al. [118]

*Original data reported as odds of developing Buruli ulcer in cases compared to noninfected controls, converted to odds of not developing Buruli ulcer in noninfected individuals compared to cases.

OR, odds ratio.

In addition, efforts should be dedicated to ensuring hygiene is maintained in areas of West Africa where Buruli ulcer is endemic. As *M*. *ulcerans* can cause infection via exposed wounds, adequate and proper wound care should be maintained to prevent infection [12,119,120]. Additionally, the regular use of bed nets and insect repellents has been associated with a lower incidence of Buruli ulcer, which may be a consequence of deterring contact with potential insect vectors [12,119]. These observations support a possible role of insects and mosquitoes in the mechanical transmission of *M*. *ulcerans*, where insect bites puncturing the skin may facilitate the colonisation and entry of the pathogen [9]. Future studies may investigate other biting insects to explore this hypothesis further and uncover any other potential vectors.

An important observation from several studies in West African countries is that education, both general and Buruli ulcer specific, is associated with a lower incidence of Buruli ulcer [118,119]. Improving access to education and educational facilities in endemic regions may be a significant and effective intervention in reducing case numbers [125,126]. More specifically, targeted educational programmes about Buruli ulcer and the causes and risks should be implemented to increase awareness of this disease and improve daily decision-making to help individuals protect themselves from contracting Buruli ulcer. This could influence whether individuals spend time in water, the protective measures they employ in these environments, and their hygiene practices to minimise the risk of environmental transmission. Early identification of lesions and disease diagnosis is an important strategy in ensuring treatment is prompt in preventing severe disease progression. Various community service and surveillance programmes have already been established throughout West Africa, which have seen increased clinical presentations of cases in early stages where treatment is more effective [127–131]. Additionally, programmes such as these could be extended in promoting community education of the various risk factors associated with Buruli ulcer to prevent infection and reduce the burden of infection in these communities.

Corresponding prevention case–control studies have not yet been conducted in Southeast Australia. However, given *M*. *ulcerans* is found in aquatic sites in endemic regions of Australia and evidence of vector transmission via mosquitoes, certain preventive measures identified by West African studies would likely be effective in preventing infection in Southeast Australia [16,26]. Specifically, reducing contact with water sources, maintaining adequate wound care, and the use of sleeved clothing and insect repellents may be effective strategies. However, evidence of mosquito vectors and mammalian reservoirs such as possums is stronger in Southeast Australia than in West Africa [8,19]. Thus, different control measures that interrupt specific transmission routes may be required in Southeast Australia. This may include epidemiological tracking of *M*. *ulcerans* among important animal reservoirs and reducing human contact with these species. Targeted mosquito control trials are planned to determine whether mosquito reductions are associated with reduced Buruli ulcer incidence in the Mornington and Bellarine peninsulas [132].

## Future directions

Research to decipher the modes of transmission of *M*. *ulcerans* is expansive and ongoing, but, ultimately, the precise transmission pathways to humans remain unclear. Aquatic environments play a key role in sustaining *M*. *ulcerans* and appear to be a major route of infection, posing challenges in rural and agricultural communities in West Africa. Vertebrates and insects can carry *M*. *ulcerans* bacilli, but whether this is a risk factor for human infection has not been adequately demonstrated. Further studies elucidating the roles of animal hosts and potential insect vectors in this environmental transmission pathway would provide valuable evidence into the more specific routes of transmission of *M*. *ulcerans* and may identify further environmental risks to humans. As there is strong evidence in Australia for mosquitoes as vectors of *M*. *ulcerans*, future research should explore this relationship further to establish vector competence and resolve the mechanism and nature of mosquito carriage of *M*. *ulcerans*, including the mode of bacterial persistence within mosquitoes [5]. Studies in West Africa are also needed to investigate transmission via mosquitoes further and compare this with findings in Australia. We suggest five key research priority areas (Box 1) that require attention to advance understanding of Buruli ulcer transmission and suggest that future studies on pathogen transmission and prevention should operate under a One Health framework. This reflects that Buruli ulcer is evidently a disease of environmental origin in both humans and mammals, and environmental disturbance is linked to outbreaks. With a vaccine not presently available, efforts to prevent infection in vulnerable communities should also be a priority, including improving access to adequate sanitation and education on the risks and causes of Buruli ulcer.

**Box 1. Buruli ulcer transmission: Current research priorities**

**Adoption of One Health frameworks**

*M*. *ulcerans* can reside in humans, animals, the environment, and other sources. However, the transmission and control of Buruli ulcer is rarely investigated through One Health frameworks. Unified approaches that concurrently monitor *M*. *ulcerans* in several sources are needed to guide environmental survey designs and studies inferring transmission using bacterial phylogenomics. Integrating prevalence and genomic information from multiple reservoirs, rather than focusing on only one, will aid in understanding transmission between them. Moreover, with evidence that Buruli ulcer incidence is linked with environmental disturbances, it is critical to assess how changes in environmental and animal health impact Buruli ulcer transmission. A better integrated understanding of disease transmission would inform policies and interventions to reduce Buruli ulcer spread.

**Establishing vector competence**

There is good evidence for an association between mosquitoes, *M*. *ulcerans*, and human Buruli ulcer cases in areas of Southeast Australia. Initial experimental studies on vector competence of local mosquitoes have been performed, but further observational and laboratory studies are required to define the precise roles that mosquitoes play as vectors in the transmission of *M*. *ulcerans* to humans. These studies should be combined with additional surveillance of mosquitoes and native possum populations to monitor these associations and perhaps build predictive mathematical models that can guide targeted mosquito control trials. In African countries, despite several large-scale insect surveys, there is minimal evidence for mosquitoes as a vector for *M*. *ulcerans*, but new vector competence studies are required to better understand the roles other invertebrates (e.g., water bugs) might play in pathogen transmission on that continent.

**Resolving transmission chains within and between animal reservoirs**

Australian native possums are reservoirs of *M*. *ulcerans* in Australia. However, the links between chains of transmission are not well established. It is not clear if *M*. *ulcerans* is transmitted from possums to other hosts or vectors, or how possums become infected. Bacterial comparative genomics and phylogeographic studies may help elucidate how *M*. *ulcerans* is spread between hosts, identifying intervention points for disease control.

**Culturing *M*. *ulcerans* from environmental samples**

Detection of *M*. *ulcerans* in the environment is challenging, with qPCR-based assays providing limited information and isolation in pure culture achieved in only one published study. It is critical to develop approaches to determine the viability (e.g., improved cultivation methods) and population structure (e.g., culture-independent genomics) of *M*. *ulcerans* in the environment. These will be important steps in standardising and accelerating Buruli ulcer research and will aid in studies aimed at resolving pathogen transmission between humans, other animals, and the environment.

**Comparing different modes of transmission between continents**

Current evidence suggests different routes of transmission between West Africa and Southeast Australia, though studies have not directly compared transmission between these two continents. Comparative research employing standardised and consistent protocols for vector competence, comparative genomics, and direct detection of *M*. *ulcerans* in complex environmental samples are needed to confirm whether transmission routes vary within and between countries and why this is so, guiding best practice for *M*. *ulcerans* prevention in all endemic locations.

**Key Learning Points**

- *M*. *ulcerans* is an environmental pathogen found predominantly in West Africa and Southeast Australia. Contamination of broken skin in these environments seems to be a major risk factor for developing Buruli ulcer.
- In Southeast Australia, mammals such as native possums are symptomatic hosts of *Mycobacterium ulcerans*, and genotyping confirms that they are part of the same transmission network as humans. Evidence for mammalian hosts in West Africa is weaker.
- In Southeast Australia, mosquitoes likely mediate transmission of *M*. *ulcerans*. There is no evidence for mosquitoes spreading Buruli ulcer in African countries, although other biting aquatic insects may play a role.
- In African countries, outbreaks of Buruli ulcer are sometimes preceded by human disturbances to the environment, creating favourable environments for *M*. *ulcerans*.

**Top Five Papers**

1. Fyfe JAM, Lavender CJ, Handasyde KA, Legione AR, O’Brien CR, Stinear TP, et al. A major role for mammals in the ecology of *Mycobacterium ulcerans*. PLoS Negl Trop Dis. 2010;4(8).
2. Lavender CJ, Fyfe JAM, Azuolas J, Brown K, Evans RN, Ray LR, et al. Risk of Buruli ulcer and detection of *Mycobacterium ulcerans* in mosquitoes in Southeastern Australia. PLoS Negl Trop Dis. 2011;5(9).
3. Wallace JR, Mangas KM, Porter JL, Marcsisin R, Pidot SJ, Howden B, et al. *Mycobacterium ulcerans* low infectious dose and mechanical transmission support insect bites and puncturing injuries in the spread of Buruli ulcer. PLoS Negl Trop Dis. 2017;11(4):e0005553.
4. Maman I, Tchacondo T, Kere AB, Beissner M, Badziklou K, Tedihou E, et al. Molecular detection of *Mycobacterium ulcerans* in the environment and its relationship with Buruli ulcer occurrence in Zio and Yoto districts of maritime region in Togo. PLoS Negl Trop Dis. 2018;12(5).
5. Degnonvi H, Fleuret S, Coudereau C, Gnimavo R, Giffon S, Yeramian E, et al. Effect of well drilling on Buruli ulcer incidence in Benin: a case-control, quantitative survey. Lancet Planet Health. 2019;3(8):e349-e56.
